# Battle of Thermopylae: 300 Spartans (natural killer cells plus obinutuzumab) versus the immortal warriors (chronic lymphocytic leukemia cells) of Xerxes’ army

**DOI:** 10.2144/fsoa-2019-0064

**Published:** 2019-11-26

**Authors:** Ricardo García-Muñoz, María-Josefa Nájera, Jesús Feliu, Judith Antón-Remírez, Enrique Ramalle-Gómara, Raquel Marín-Gorricho, Raisa Peralta, Elena Gutiérrez-Gamarra, Jessica Nuñez-Rodriguez, Ricardo Zafra-Morales, Lorea Aguinaga, María-Jose Nebot-Villacampa, Prisma-Monserrat Hernandez-Pérez, Giovanna Farfán-Quiroga, Carlos Panizo, Elena Domínguez-Garrido

**Affiliations:** 1 Department of Hematology, San Pedro Hospital, 26006 Logroño, La Rioja, Spain; 2 Department of Physical Medicine & Rehabilitation, Navarra Hospital Complex, Pamplona, Spain; 3 Department of Epidemiology, La Rioja Regional Authority, Logroño, La Rioja, Spain; 4 Department of Pharmacy, San Pedro Hospital, Logroño, La Rioja, Spain; 5 Department of Hematology, Poniente Hospital, Almería, Andalucía, Spain; 6 Department of Hematology, Centre Hospitalier Universitaire (CHU), Bordeaux, France; 7 Hematology Department, University Clinic of Navarra, Pamplona, Navarra, Spain. Lymphoproliferative Group, Navarran Institute for Health Research (IDISNA), Pamplona, Navarra, Spain; 8 Molecular Diagnostic Unit, Rioja Health Foundation, Logroño, La Rioja, Spain

**Keywords:** ADCC, chronic lymphocytic leukemia, follicular lymphoma, GA101, ibrutinib, NK cells, obinutuzumab, subcutaneous rituximab

## Abstract

**Aim:**

To analyze the effects of subcutaneous or intravenous rituximab + lymphokine-activated killer cells, obinutuzumab or ibrutinib on natural killer (NK) cell levels in chronic lymphocytic leukemia and follicular lymphoma patients.

**Patients & methods:**

The distribution of peripheral blood NK cells of 31 patients was analyzed by flow cytometry.

**Results:**

We detected a decrease of NK cells in peripheral blood below normal range after obinutuzumab treatment. During maintenance treatment with subcutaneous rituximab, an NK cell reduction was less pronounced than after intravenous rituximab treatment, despite lymphokine-activated killer cell infusions.

**Conclusion:**

After one dose of obinutuzumab, each NK cell in peripheral blood destroys 25 leukemic cells.

## Lay abstract

The standard treatment of chronic lymphocytic leukemia and follicular lymphoma is chemotherapy in combination with anti-CD20 monoclonal antibodies, resulting in the destruction of the immune system, or a ‘Kamikaze effect’. Unfortunately, immunotherapy with rituximab or obinutuzumab may be of limited efficacy when the immunological system is overwhelmed by abundant tumor cells or is diminished by chemotherapy, which eliminates effector immune cells such as natural killer cells before they would be able to kill the whole tumor. Hence, it is important to measure the number of immune cells to ensure that during the encounter of effector cells with tumor cells, sufficient ‘warriors’ can win the battle against the tumor. Otherwise, something akin to the Battle of Thermopylae can happen where a limited number of Spartan warriors faced a huge army and were defeated in the end.

In humans, natural killer (NK) cells seem best suited for surveying the blood for transformed cells and also abound in bone marrow and lymph nodes. These cells (Spartan warriors) are strategically located to monitor and destroy tumor cells (Xerxes’ army) before they grow [1]. The first molecularly defined activation receptor on NK cells is CD16, through which NK cells mediate antibody-dependent cytotoxicity (ADCC) against IgG-coated targets. Obinutuzumab (GA101) is a glycoengineered, type II anti-CD20 molecule with less complement-dependent cytotoxicity (CDC) than type I anti-CD20 rituximab though greater ADCC [2].

Interestingly, although GA101 seems to be superior to rituximab in inducing direct cell death and ADCC both *in vitro* [3] and in some clinical trials [4,5], in the early randomized Phase II GAUSS study, single-agent comparison between obinutuzumab and rituximab in patients with relapsed indolent lymphoma did not generate encouraging results [6]. This trial is very interesting because it compares type 1 and 2 monoclonal antibodies anti-CD20 without any combining drug; however, the real effect of immunotherapy alone, in combination with a robust immune system, remains concealed in chronic lymphocytic leukemia (CLL). Others, us included, have demonstrated that GA101 monotherapy induces NK cell depletion in the peripheral blood of patients with CLL [7,8]. Previously, we demonstrated that the percentage of NK cells in lymphokine-activated killer (LAK) cells from patients with follicular lymphoma (FL) correlated with ADCC against CD20^+^ lymphoma B cell lines *in vitro* [9]. CD56 is restricted to NK cells and a subpopulation of T cells. In LAK cells, 65% of the expanded cells express CD56 and we found that the percentage of NK cells (CD56^+^) among LAK cells was correlated with rituximab and GA101-induced ADCC [9]. *In vitro*, GA101 plus LAK cells (enriched with NK cells) induced stronger ADCC than rituximab plus LAK cells [9]. We hypothesize that levels of NK cells in peripheral blood from patients with CLL treated with GA101 correlate with leukemic tumor destruction induced by an NK-ADCC effect.

To evaluate these effects, we analyzed the distribution of CD56^+^ NK cells and CD19^+^ leukemic B cells in the peripheral blood of 12 patients with CLL who were treated with GA101 monotherapy. We also explored ten patients with FL who had received subcutaneous rituximab or intravenous rituximab plus LAK cells, and evaluated the kinetics of NK cells in this population. In a similar way, we also analyzed nine patients with CLL treated with the B-cell receptor inhibitor ibrutinib.

## CLL & FL patients

### Obinutuzumab (GA101)

Twelve patients (six previously untreated and six refractory/relapsed) diagnosed with CLL or small lymphocytic lymphoma and treated with GA101 were studied. GA101 monotherapy was administered intravenously in all patients in cycle 1 on day 1 (100 mg) and day 2 (900 mg) following the manufacturer's recommendations.

The distribution of peripheral blood lymphocytes was examined before the first dose of GA101 and 24 or 72 h after the first complete 1000 mg dose (cycle 1, day 2).

### Subcutaneous rituximab & intravenous rituximab plus LAK cells

FL patients included in the analysis were treated with the same procedure indicated in clinical trials MABRELLA [10] and LFNK [11]. LAK cells were generated as described previously [9].

In brief, five patients with FL received at least one full dose of intravenous rituximab during R-CHOP (rituximab–cyclophosphamide–doxorubicin–vincristine–prednisone) and maintenance therapy (1400 mg of subcutaneous rituximab once every 2 months for 12 cycles, accordingly with the MABRELLA trial [10]). The other five patients who received R-CHOP and 24 months of bimonthly doses of 375 mg/m^2^ of intravenous rituximab maintenance therapy also received >5 × 10^8^ LAK cells in the pair cycles, accordingly with the LFNK trial [11]. We determined NK cell counts in peripheral blood by flow cytometry using anti-CD56 monoclonal antibodies.

### Ibrutinib

To describe the redistribution of NK cells during a noncytotoxic CLL treatment, we also studied nine patients diagnosed with CLL and treated with ibrutinib (480 mg daily). The distribution of NK cells in these patients was examined prior to treatment (baseline) and after 30 days of continuous treatment.

## Material & methods

Cells were counted by flow cytometry with monoclonal antibodies against CD19 and CD56. Flow count™ fluorospheres (Beckman Coulter, CA, USA) were used to determine absolute counts in 100,000 lymphocytes, according to the manufacturer's instructions.

The Wilcoxon and/or sign test (paired samples) and Mann–Whitney U test (random independent samples) were used to estimate the statistical significance of the differences observed.

A multiple linear regression model was used to adjust the effect of the base line count of NK cells and the number of tumor cells on tumor destruction. We applied the linear regression model because the assumptions of the model are fulfilled, since the Shapiro–Wilk test on the residuals of the model gave a nonsignificant value. We also used Spearman's Rank-Order Correlation test to evaluate possible correlations between NK cell counts and tumor burden destruction (baseline CD19 B cells - CD19 B cells after 1000 mg of obinutuzumab = tumor burden destruction). Statistical analysis was performed using Social Science Statistics software [12]; p < 0.05 was considered statistically significant.

The analysis was performed according to specific research ethics protocols and after approval of the local institutional review board. All patients gave their consent to use anonymized data.

## Results

### CLL treated with GA101 (‘Thermopylae battle effect’)

GA101 is approved for the treatment of patients with CLL and FL, and activates NK cells through CD16; notably CD16 does not require co-activating signals to trigger an NK cell response.

All patients in the study received at least one complete dose of 1000 mg of GA101; eight patients completed their treatment (six cycles of GA101) and immunological characteristics were published previously [8]. At the time of submission, 11 of the 12 patients are alive. Clinical and immunological characteristics of the patients are summarized in Tables 1 and 2.

**Table 1.** Immunological characteristics of 12 chronic lymphocytic leukemia patients treated with obinutuzumab.

CD56^+^ NK cells (effector cells/ml)	Baseline	After obinutuzumab	CD19 (target cells/ml) Baseline	After obinutuzumab	Tumor destruction in PB
Patient 1	**314**	24	204,052	170,355	**33,697**
Patient 2	**396**	60	229	8	**221**
Patient 3	**1923**	36	62,338	357	**61,981**
Patient 4	305	ND	93,435	ND	ND
Patient 10	**221**	17	61,495	22,836	**38,659**
Patient 12	**147**	6	492	4	**488**
Patient 5	**67**	1	214	248	**0**
Patient 6	**91**	10	115	419	**0**
Patient 7	343	ND	12283	ND	ND
Patient 8	**483**	10	47,067	15,383	**31,684**
Patient 9	**235**	1	6688	309	**6379**
Patient 11	**284**	76	68,831	17447	**51,384**

The bold details are to indicate the values used in the statistical analysis (paired samples).

Cells were counted by flow cytometry with monoclonal antibodies against CD56 and CD19. CD56^+^ cells represent ‘classic warriors’ such as NK cells and T cells in PB of CLL patients. These CD56^+^ cells represent effector cells. CD19^+^ cells represent target cells and represent leukemic CLL B cells and normal B cells in the blood of CLL patients. Tumor destruction in PB = baseline target cells - target cells detected after one 1000 mg dose of obinutuzumab. Both CD19 counts in tumor destruction in PB are used to detect the association between effectors (NK cell count) and tumor destruction in PB using a Rho Spearman's (correlation) test. (rs = 0.63222, p[2-tailed] = 0.049). A linear regression model analyzing the relationship between the baseline effectors (CD56^+^ NK cells) and target (CD19^+^ leukemic CLL B cells) destruction shows that on average, 25 target CD19^+^ cells are destroyed by each effector CD56^+^ cell (p = 0.03777). The regression equation is: number of targets destroyed = 3670.4 + 24.8 * NK + 0.187 * baseline tumor, with R2 of 0.67.

CLL: Chronic lymphocytic leukemia; ND: Not done; NK: Natural killer; PB: Peripheral blood.

**Table 2.** Immunological characteristics of 12 chronic lymphocytic leukemia patients treated with obinutuzumab.

Patient ID	Cytogenetics (FISH)	*IGHV* status	Treatment	Binet stage/bone marrow	Age	Gender	Response to obinutuzumab	Status and follow-up since start of obinutuzumab	Previous treatments	Salvage therapy post-obinutuzumab
Patient 1	Deletion 13q	Mutated	Untreated	C	61	F	CR	Relapse at 23 months	None	Ibrutinib
Patient 2	Trisomy 12/deletion 11q	Unmutated	Untreated	B	84	M	CR	Relapse at 38 months	None	None
Patient 3	Normal	Unmutated	Untreated	B	76	F	CR	Remain CR after 28 months	None	None
Patient 4	Deletion 13q homozygous	Mutated	Untreated	C	67	M	CR	Relapse at 27 months	None	None
Patient 10	Trisomy 12	Unmutated	Untreated	B	69	F	In treatment	In treatment	None	None
Patient 12	Normal	Mutated	Untreated	B (BM 48%)	68	F	In treatment	In treatment	None	None
Patient 5	Deletion 11q	Unmutated	R/R	C (BM 50%)	76	F	PR	Relapse at 17 months	R monotherapy, R-CLB	Ibrutinib
Patient 6	Trisomy 12 deletion 13q	ND	R/R	C (BM 30%)	71	F	PR	Remain in CR after 28 months	R monotherapy, R-HDMP	None
Patient 7	Normal	Mutated	R/R	C	66	F	PR	Relapse after 22 months	R-HDMP, BR	Ibrutinib
Patient 8	Deletion 11q	Unmutated	R/R	C	88	M	PD (exitus; cerebral hemorrhage)	Progressive disease	R-CHOP, R monotherapy, R-CLB	None
Patient 9	Deletion 11q	ND	R/R	C	65	F	PR	4 cycles of obinutuzumab (PR)	Fludarabine, FMD, Alemtuzumab, R-FC plus allo-SCT	Ibrutinib
Patient 11	Normal	ND	R/R	C	81	M	CR	Remain in CR at 9 months	BR, R monotherapy	None

B: Bendamustine; BM: Bone marrow; BR: Bendamustine–Rituximab; CHOP: Cyclophosphamide–doxorubicin–vincristine–prednisone; CLB: Chlorambucil; CR: Complete response; FC: Fludarabine cyclophosphamide; FMD: Fludarabine–mitoxantrone–dexamethasone; HDMP: High-dose methylprednisolone; IGHV: Immunoglobulin heavy chain gene; ND: Not done; PD: Progressive disease; PR: Partial response; R: Rituximab; R/R: Relapsed or refractory; Stage C: Anemia <10 g/dl and/or thrombocytopenia <100,000mm^3^.

#### Immunological analysis

**Figure 1.**
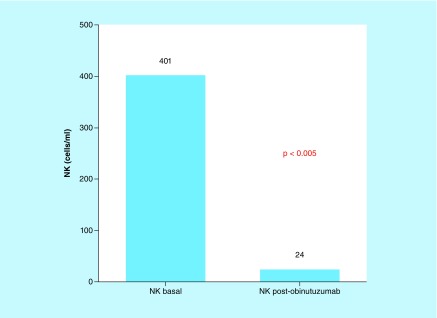
NK lymphocytes before and after obinutuzumab. Obinutuzumab induces depletion of natural killer cells in patients with chronic lymphocytic leukemia (Mann–Whitney U test). In chronic lymphocytic leukemia, type 2 anti-CD20 monoclonal antibody therapy such as obinutuzumab administered intravenously, detects both effector cells (NK cells) and target cells (leukemia cells) in the blood. This figure represents the effect of obinutuzumab in effector cells (NK cells) after one 1000 mg dose of obinutuzumab. Baseline effector cells (402 NK cells/μl) and post-treatment effector cells are represented (24 NK cells/μl). NK: Natural killer cells.

After the first complete 1000 mg GA101 dose (24–72 h after cycle 1, day 2), NK cells significantly decreased in peripheral blood compared with base line counts prior to therapy (402 cells/μl baseline vs 24 cells/μl postinfusion, p ≤ 0.0001; Figure 1). This reduction was observed independently of NK cell levels before therapy (p ≤ 0.05).

**Figure 2.**
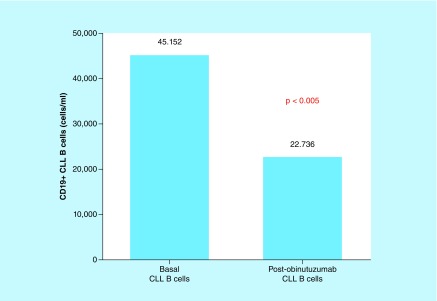
CD19+ CLL B cells before and after obinutumab. Wilcoxon signed rank test: leukemic burden destruction after the first dose of obinutuzumab depends on natural killer-antibody-dependent cytotoxicity (log scale). In CLL, type 2 anti-CD20 monoclonal antibody therapy such as obinutuzumab administered intravenously encounters and interacts with both effector cells (natural killer cells) and target cells (leukemia cells) in the blood. This figure represents the effect of obinutuzumab on target cells (CLL B cells). Note that this effect is induced by antibody-dependent cytotoxicity of effector cells (natural killer cells) redirected by obinutuzumab. Baseline target cells (45,152 CLL B cells/μl) and post-treatment target cells are represented (22,736 CLL B cells/μl). CLL: Chronic lymphocytic leukemia.

No statistically significant differences in the B-cell compartment were detected between base line counts and after 24–72 h (46,437 cells/μl baseline vs 22,449 cells/μl 24–72 h postinfusion; p = 0.222). However, a significant decrease in the B-cell count was detected when paired samples were analyzed (45,152 cells/μl baseline vs 22,736 cells/μl; p ≤ 0.0164), suggesting that the reduction of CLL B cells depends not only on the anti-CD20 monoclonal antibody GA101 (Figure 2).

This reduction was specific and independent of the decrease in white blood cell counts; essentially, some of the study patients had normal baseline levels of leukocytes and low leukemic burden in peripheral blood (Table 1, patients 2, 5, 6 and 12),

#### Statistical analysis

To confirm that the CLL B-cell reduction in peripheral blood is brought about by NK cells, and not only mediated by the monoclonal antibody, we used linear regression. The multiple linear regression model, which analyzes the relationship between the baseline NK number and leukemic CLL B-cell destruction, shows that, on average, 25 leukemic cells are destroyed by each NK cell (p = 0.0377). The regression equation is as follows: number of cells destroyed = 3670.4 + 24.8 * NK + 0.187 * baseline tumor, with an R2 of 0.67.

The Wilcoxon signed rank test (paired samples) was used to compare the baseline values and 24 or 72 h in the same patients.

There is a statistical association between the number of NK cells and the destruction of the leukemic CLL B cells (p = 0.0377), once adjusted for the baseline size of the tumor. On average, 25 tumor cells are destroyed per NK.

Rho Spearman's (correlation) test confirmed the association between the two variables (baseline NK cell count and tumor destruction in peripheral blood) to be statistically significant (r_s_ = 0.63222; p[2-tailed] = 0.049).

### Subcutaneous rituximab or intravenous rituximab plus LAKs cells in FL patients & their effects on blood NK cells

Rituximab exerts its antilymphoma or leukemia activity by binding to CD20 on the surface of B cells, including the malignant clones. It reaches its target via the blood circulation. Rituximab serum trough concentration reflects the degree of target site saturation on B cells; ensuring that serum concentrations are at least as high after subcutaneous administration can therefore be expected to result in at least the same degree of target site saturation – and hence at least the same efficacy [13]. Both the subcutaneous and intravenous formulations deliver identical rituximab, and the similar overall responses, complete responses and safety profiles suggest that subcutaneous administration of rituximab does not compromise the antilymphoma activity of rituximab when given with chemotherapy or in the maintenance treatment [13–15]. Rituximab can induce stronger CDC and ADCC. In contrast to type I, type II anti-CD20 obinutuzumab induces stronger ADCC through NK cells but not CDC. Unfortunately, it is very difficult to demonstrate the individual contribution of ADCC or CDC during rituximab treatment on patients with lymphoma or leukemia.

In an attempt to clarify the possible confusing influence of CDC from rituximab and the distribution of NK cells during anti-CD20 treatment, we retrospectively analyzed ten patients with FL treated with subcutaneous rituximab (n = 5) and intravenous rituximab (n = 5) plus LAK cells (enriched with CD56^+^ NK and T cells) who received R-CHOP as induction and then received maintenance therapy with rituximab. We selected patients without blood infiltration in order to avoid the confusing factor of target cells (leukemic B cells) and because subcutaneous rituximab travels first to the lymphatic system (tissue of secondary lymphoid organs) and IV rituximab plus LAK cells (enriched in NK cells) represent the primed effectors (NK plus anti-CD20) in blood that migrate to other sites to exert their effect. Analysis after R-CHOP and during rituximab maintenance ensures that low or inexistent CD19 B cells (target) masquerade the results.

#### Results & analysis

Baseline counts of NK cells in patients treated with R-CHOP were 163 NK cells/μl (52–854 NK cells/μl). After six cycles of R-CHOP, NK cell counts diminished markedly to 84 NK cells/μl (27–291 NK cells/μl) below normal reference value (95–640 NK cells/μl). During maintenance treatment with six doses of rituximab (12 months after R-CHOP), NK cell counts increased progressively to reach 222 NK cells/μl (117–498 NK cells/μl). Interestingly, patients who received subcutaneous rituximab exhibited significantly more NK cells (177 NK cells/μl [95–291] all within normal reference limits) than patients receiving intravenous rituximab (66 NK cells/μl [27–73] after induction with R-CHOP [p < 0.05] and after three doses of rituximab of maintenance [p < 0.05] [329 NK cells/μl (184–423) vs 132 NK cells/μl (74–217) 6–8 months after R-CHOP]). However, these differences disappeared after six doses of maintenance with rituximab (12–14 months post-R-CHOP; p > 0.05). Notably, recovery of NK cells was faster in patients who received subcutaneous rituximab than intravenous rituximab plus LAK cells throughout the first 6–8 months after induction of R-CHOP (Tables 3a and b).

**Table 3a.** Treatment with rituximab-cyclophosphamide–doxorubicin–vincristine–prednisone induces a ‘Kamikaze effect’ with a reduction of natural killer cells.

All patients	NK pre-R-CHOP	NK after-R-CHOP	NK (1 Ritux)	NK (3 Ritux)	NK (6 Ritux)
	52	73	176	217	215
	262	27	188	176	132
	163	65	103	85	230
	**256**	66	95	132	195
	**135**	73	52	74	117
	**108**	**95**	**180**	**329**	**230**
	**854**	**124**	**232**	**184**	**117**
		**230**	**162**	**333**	**498**
		**177**	**341**	**233**	**228**
		**291**		**423**	**369**
Xme	163	84	176	201	222
(X_min_–X_max_)	(52–854)	(27–291)	(52–341)	(74–423)	(117–498)

Bold details are only to differentiate patients that receive intravenous rituximab from the patients with subcutaneous rituximab.

Effector cells (NK cells) are slightly below normal reference values (87 NK cells/ml), improved however, during maintenance with rituximab effector cells (NK cells). Rituximab maintenance therapy causes the encounter of effector cells (NK cells) with monoclonal antibody, but without target cells (leukemia or lymphoma blood cells), in blood compartment. This may be a model of the distribution of NK cells in the presence of anti-CD20 antibodies without target cells and may help to explain the phenomenon in which some patients with deep responses to obinutuzumab recover NK cell counts when nontumoral cells in blood or secondary lymphoid organs may be located.

NK: Natural killer; R-CHOP: Rituximab–cyclophosphamide–doxorubicin–vincristine–prednisone; X_max_: Maximal value; Xme: Median X value; X_min_: Minimun value.

**Table 3b.** Unexpectedly, SC rituximab did not induce diminution of natural killer cell levels in sharp difference to intravenous rituximab, even when infusions of lymphokine-activated killer cells (enriched with ‘classical warriors’ such as CD56^+^ natural killer and γδ T cells’ in these patients.

Protocol	U-Mann p < 0.05	N	U-Mann p < 0.05	NS p > 0.05	Age	Gender	Ann Arbor stage	IV R-CHOP+ LAKs	Status	Second line
**LFNK**	**NK (after R-CHOP)**	**NK (1 Ritux IV)**	**NK (3 Ritux IV)**	**NK (6 Ritux IV)**						
	73	176	217	215	52	**Female**	**IV-B**	6	**CR**	
	27	188	176	132	49	Female	IV-B	6	CR	Regional radiotherapy
	65	103	85	230	64	Male	IV-B	6	CR	
	66	95	132	195	64	Female	IV-B (spleen)	6	CR	
	73	52	74	117	80	Female	III-A	6	CR	
Xme	66	103	132	195						
(X_min_–X_max_)	(27–73)	(52–188)	(74–217)	(117–230)						
		NS	Sign test p = 0.025	Sign test p = 0.025						
MABRELLA	**NK(after R-CHOP)**	**NK (1 sc Ritux)**	**NK (3 sc Ritux)**	**NK (6 sc Ritux)**	**Age**	**Gender**	**Ann Arbor stage**	**R-CHOP+ sc R main**	**Status**	**Second line**
	**95**	**ND**	**329**	**230**	78	**Female**	**III-A**	8	**CR**	
	**124**	**180**	**184**	**117**	83	Female	IV-B	6	CR	
	**230**	**232**	**333**	**498**	68	Male	IV-A	6	R/R	R-ESHAP + ASCT
	**177**	**162**	**233**	**228**	67	Female	IV-B	8	CR	
	**291**	**341**	**423**	**369**	79	Female	IV-A	6	CR	
Xme	177	206	329	230						
(X_min_–X_max_)	(95–291)	(162–341)	(184–423)	(117–498)						
		ND	Sign test p = 0.025	Sign test p = 0.179						

Bold details are to differentiate patients with subcutaneous (MABRELLA) or intravenous rituximab (LFNK).

This may represent a model in which immediate encounter of NK cells with anti-CD20 monoclonal antibodies in blood may prepare them to fight and migrate to secondary lymphoid organs ‘searching the enemy’. In contrast, SC rituximab unmasks enemies in secondary lymphoid organs to be recognized by ‘classical warriors’ such as NK and γδ T cells when they perform their normal immunosurveillance and discover tumoral cells coated with monoclonal antibodies.

ASCT: Autologous peripheral blood stem cell transplant; CHOP: Cyclophosphamide–doxorubicin–vincristine–prednisone; ESHAP: Etoposide-solumedrol-ARA-C-cisplatin; LAK: Lymphokine-activated killer; ND: Not done; NK: Natural killer; NS: Not significant; R: Rituximab; R-ESHAP: Rituximab-Etoposide-solumedrol-ARA-C-Cisplatin; CR: Complete response; R/R: Relapsed or refractory; SC: Subcutaneous; X_max_: Maximal value; X_me_: Median X value; X_min_: Minimum X value.

### CLL treated with ibrutinib (‘sleeping beauty effect’)

**Figure 3.**
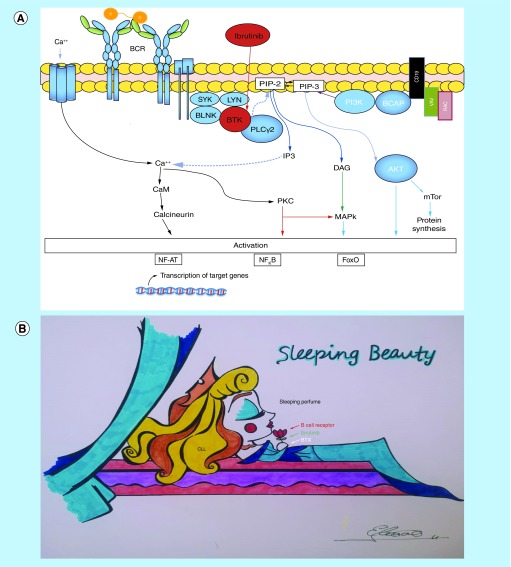
Sleeping Beauty effect. **(A)** Ibrutinib is a Bruton's tyrosine kinase inhibitor, which increases the apoptotic susceptibility of malignant lymphocytes and also causes tissue redistribution of tissue-resident chronic lymphocytic leukemia cells into the blood with rapid shrinkage of the lymph nodes. Bruton's tyrosine kinase is essential for chemokine-mediated homing and adhesion of B cells, and for the activation of several pathways, contributing to chronic lymphocytic leukemia cell survival, including AKT, and NF-κB pathways. Combinations such as ibrutinib and obinutuzumab or ibrutinib and venetoclax may be safe and effective treatment options. **(B)** ‘The Sleeping Beauty effect’. The drawing exemplifies a dormant CLL cell breathing the scent of the sleep spell when ibrutinib inhibits Bruton's tyrosine kinase. CLL: Chronic lymphocytic leukemia.

Ibrutinib covalently inhibits Bruton tyrosine kinase and targets B-cell receptor signaling, while modifying CLL microenvironment. Unfortunately, ibrutinib induces a ‘sleeping beauty effect’ [16], characterized first by transient lymphocytosis with unmaintained remission after discontinuation of therapy in CLL patients [17] and second by the inhibition of antilymphoma T cells and NK function [18]. Similar to the witch's spell in sleeping beauty, treatment with ibrutinib makes the CLL cells ‘fall asleep’, which then leave the lymph nodes to float asleep through peripheral blood (lymphocytosis) and also to the immune system by decreasing ADCC induced by rituximab and obinutuzumab (Figure 3).

#### Results & analysis

Patients treated with ibrutinib showed an increment of the NK cell count after 30 days of treatment in comparison with basal pretreatment NK cell count (baseline 311 NK cells/μl vs 410 NK cells/μl after treatment; p = 0.317). These findings suggest that the ‘sleeping beauty effect’ also affects peripheral blood NK cells in patients with CLL (Table 4).

**Table 4.** As expected, patients with immediate ibrutinib effects showed no significant changes in the natural killer cell counts after 30 days of treatment (p = 0.317) in concordance with their ‘sleeping beauty effect’ in both leukemic cells (data not shown) and natural killer cells (inhibit their action).

Treatment	Patient	Cytogenetics (FISH)	*IGHV* status	Age	Gender	Baseline NK/ml	After 30 days NK/ml	Ibrutinib previous lines
**Ibrutinib**	1	trisomy12/ del17p	Unmutated	78	Female	283	655	2
	2	deletion 13q	Unmutated	67	Male	1008	623	1
	3	deletion 11q/del 17p	Mutated	75	Male	101	105	3
	4	deletion 11q/del 13q	Unmutated	83	Male	635	538	1
	5	deletion 11q/del 13q	Unmutated	61	Male	363	688	2
	6	deletion 17p	Unmutated	43	Male	442	387	3
	7	Normal	Mutated	67	Female	116	211	4
	8 (dead)	deletion 11q	ND	77	Female	73	213	2
	9	deletion 17p	Mutated	62	Female	340	410	2

This may be a model in which distribution of NK cells is not altered because they are ‘sleeping’ and their immunosurveillance is too compromised to recognize leukemic cells. Interestingly, type 2 anti-CD20 antibodies but not type 1 anti-CD20 antibodies may put NK cells to sleep to ‘restore’ their immunosurveillance work.

IGHV: Immunoglobulin heavy chain gene; ND: Not done; NK: Natural killer.

## Discussion

Human NK cells can be divided into two subsets based on the expression of CD16 and the adhesion molecule CD56. CD56^dim^ NK cells represent >90% of the peripheral blood NK cells, are CD16^+^KIR^+^, and display greater cytotoxicity [19]. This clue is important to understand that anti-CD20 monoclonal antibodies administered intravenously find both effector cells (NK cells) and target cells (leukemia or lymphoma cells). When administered intravenously, rituximab and GA101 primed NK cells to kill via ADCC through CD16 receptor (GA101 primed NK cells more efficiently than rituximab), in other words, prepare the effector arm. These anti-CD20-armed NK cells may destroy target cells *in situ* (blood) or migrate through blood and lymph to secondary lymphoid organs. We describe an immediate diminution of blood NK cell counts after the first dose of GA101, suggesting that both the mechanism of destruction of leukemia cells in the blood (*in situ*) and the migration of NK cells from the blood to other lymphoid tissues may play a role in this decrease of NK counts in the blood.

This reduction persists during the whole treatment with GA101 [8]. Interestingly, the two patients with NK cell count below 100 cells/μl were unable to destroy a small quantity of CLL cells, for this reason we suggest that monoclonal antibody GA101 alone with very low NK cell count is not as effective. If GA101 without NK cells had activity, this anti-CD20 monoclonal antibody should destroy that reduced blood tumor burden. In contrast, CD56^bright^ NK cells show greater proliferative potential, greater cytokine production and are the principal NK-cell population in secondary lymphoid organs [19]. NK-cell progenitors in the lymph node that are CD56^bright^ develop through a series of intermediaries into CD56^dim^ cells [19]. It is not clear whether the CD56^dim^ cells in the blood represent an even more immature cell stage or a separate population [19]. This implies that if GA101 coats the target CLL leukemic cells, these coated B cells could be destroyed in blood by NK cells (CD56^dim^) or in bone marrow and secondary lymphoid organs where low cytotoxic NK-cell populations (CD56^bright^) reside as a mechanism of immunosurveillance.

Along the same lines, recently published findings indicate that tissue (presumably low cytotoxic CD56^bright^ NK cells) or blood NK-cell count (presumably a highly cytotoxic CD56^dim^ NK-cell population) correlates with outcome in patients with FL and diffuse large B-cell lymphoma treated with R-CHOP or GA101-CHOP [20]. Nevertheless, despite the small number of patients, our findings represent the collision (*in vivo*) in the blood of the effectors (NK cells) and targets (leukemic CLL cells) during GA101 administration in patients with CLL. In addition, this clash in the blood of NK cells and leukemic CLL cells is not influenced by CDC from rituximab.

Unexpectedly, subcutaneous administration of anti-CD20 preserved NK cells in blood in contrast to the intravenous route, despite LAK cell infusions in the absence of target blood cells such as neoplastic or normal B cells. In contrast, intravenous administration of anti-CD20 antibodies can prime high cytotoxic blood NK cells to both kill blood targets or to migrate to secondary lymphoid organs but it is unknown if intravenous administration may also induce differentiation of blood NK cells (CD56^dim^ CD16^+^) to tissue NK cells (CD56^bright^). GA101 immunotherapy might behave in a similar manner as rituximab during maintenance therapy. Attractively, in a comparable form, intravenous administration of glycoengineered type II anti-CD20 with greater ADCC (without CDC) such as GA101 may also prime NK cells to kill blood targets in concordance with our findings, in which very low levels of NK cells (24 NK cells/μl [18–498]) were detected after the first 1000-mg dose of treatment. In sharp difference with maintenance with rituximab in FL, in the case of CLL patients, several interactions between effectors (NK cells plus GA101) and targets (leukemic CLL B cells) occur. Importantly, anti-CD20 antibodies activate NK cells through CD16, and CD16 does not require co-activating signals to trigger NK-cell responses [21,22]. GA101 plus chlorambucil (Clb) demonstrated a greater effect on progression-free and overall survivals in contrast with rituximab plus Clb in patients with CLL. Undetectable minimal residual disease in peripheral blood at the end of treatment was significantly more common in patients receiving GA101-Clb than in those receiving R-Clb (35.8 vs 3.3%, respectively) [23]. Intriguingly, ibrutinib plus GA101 is an efficacious and safe chemotherapy-free combination treatment in previously untreated patients with CLL [24]; however, it is not clear whether this effective but expensive combination (GA101-ibrutinib) is superior to other classical strategies such as sequential treatment GA101 (± Clb) and rescue with ibrutinib monotherapy as second-line treatment (also a potentially chemotherapy-free strategy if Clb is avoided). It is unknown if ibrutinib's ‘sleeping beauty effect’ and inhibition of NK cells function may impair the efficacy of NK-ADCC induced by GA101 in this combination [18]. Minimal residual disease status is a known predictor of progression-free survival. Therefore, several novel combinations with anti-CD20 monoclonal antibodies intend to demonstrate deep remission rates with undetectable minimal residual disease.

**Figure 4.**
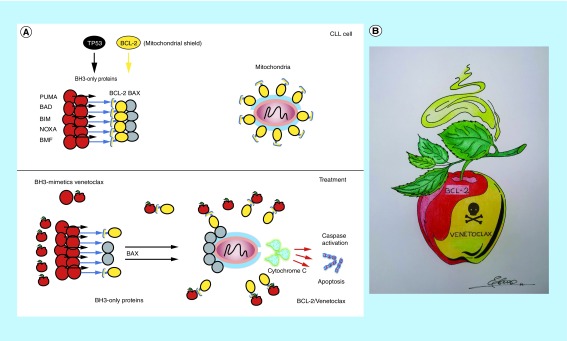
Snow White effect. **(A)** Venetoclax mimics BH3-only proteins, the native ligands of BCL-2 and apoptosis activators, by binding to the hydrophobic groove of BCL-2 proteins, thereby repressing BCL-2 activity and restoring apoptotic processes in tumor cells. Venetoclax is an effective treatment option, even in high-risk patients with chronic lymphocytic leukemia. BH3-only proapoptotic proteins favor the activation of the BAX protein, which creates pores in the mitochondria so that the cytochrome C protein is secreted, and apoptosis is triggered. BCL-2 protein inhibits both proapoptotic proteins BH3-only and BAX, preventing apoptosis. Venetoclax (poisoned apple) simulates an increase in proaptotic proteins causing BCL-2 to bind to venetoclax, leaving free proaptotic proteins that induce membrane permeability and cytochrome C output. **(B)** ‘The Snow White effect’. The drawing represents the poisoned apple of venetoclax, which binds to BCL inhibiting its protective function, promoting the apoptosis of tumor cells. BCL-2: B cell lymphoma 2.

Venetoclax, a BH3-mimetic drug with antineoplastic activity via ‘simulating’ a cellular antiapoptotic pathway (‘snow white effect’) [16], is a potent, oral BCL-2 inhibitor that has demonstrated significant activity as monotherapy and in combination with rituximab and GA101. BCL-2 is an antiapoptotic molecule that functions as a shield against proteins that favor cell death. Similar to an apple that seems healthy but is poisoned, BH3 mimics the ‘poisonous’ BCL-2 by blocking its protective function by directly binding to its BH3 domain, favoring the death of tumor cells that depend on the increased production of BCL-2 (Figure 4). Remarkably, the combination of rituximab or GA101 with venetocax was associated with longer progression-free survival and undetectable minimal residual disease [25,26]. Intriguingly, both ibrutinib and venetoclax induce a significant increase in the frequency of γδ T cells [27], which are cytotoxic cells that share several NK-cell features such as CD16 expression and ADCC. In a very recent report, Hoeres *et al.* demonstrated that both T and NK cells contribute to GA101-induced ADCC in an elegant and interestingly basic study [28].

**Figure 5.**
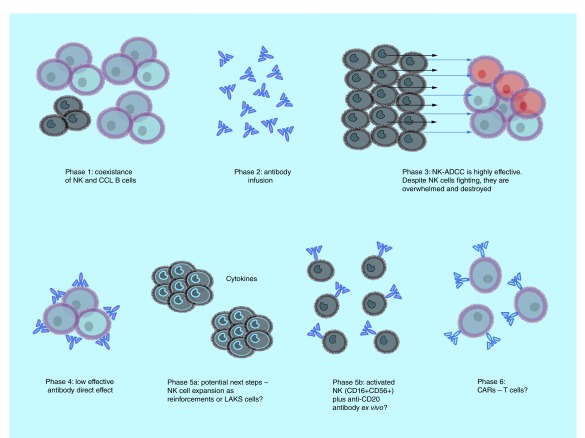
Immunological effects of obinutuzumab treatment and possible strategies to improve its function. Obinutuzumab administered intravenously unites effector cells (NK cells) and target cells (leukemia cells), and forces them to fight. This collision (*in vivo*) in the blood of the effectors (NK and γδ T cells) and target chronic lymphocytic leukemia B cells is not influenced by complement-dependent cytotoxicity and may represent a real-time antibody-dependent cytotoxicity. Activated and expanded ‘classical warriors’ such as NK and γδ T cells that are armed with CD16 plus glycoengineered antibodies may constitute a safe and low-cost therapeutical alternative to ‘Frankenstein cell therapy’ with chimeric antigen receptor T cells. ADCC: Antibody-dependent cytotoxicity; CCL: Chronic lymphocytic leukemia; NK: Natural killer.

**Figure 6.**
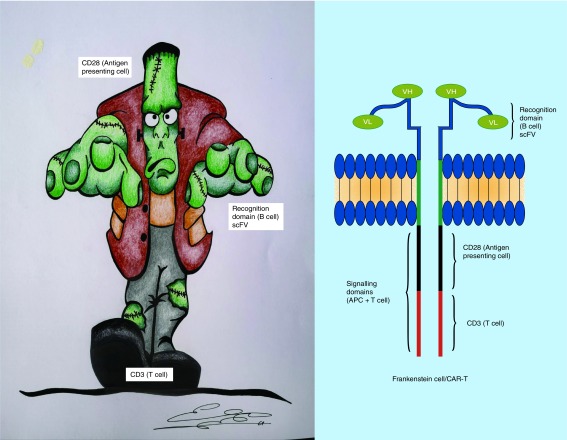
Chimeric antigen receptor T cells therapy (Frankenstein cell therapy). Genetic engineering has created a new type of T cell (Frankenstein T cell) with the generation of ‘chimeric antigen receptors’ in which a piece of B cell (single chain variable fragment or scFV) is joined with a piece of T cell (CD3ζ) and with other pieces of antigen-presenting cells (CD28 or 4-1BB). This promising new cell has the ability to destroy tumors as a rescue therapy when tumor cells are refractory to several effective treatments, but has important toxicity. scFV: Single chain variable fragment.

Numerous next-generation antibodies have been tested in the treatment of patients with lymphoma but were abandoned because they were neither more active than rituximab nor effective in the setting of rituximab resistance. Although patients with FL and CLL now have another active monoclonal antibody with GA101, prolonging patient survival with more effective and less toxic therapies remains challenging. Even with exciting new immune cell therapy such as engineered T cells expressing chimeric antigen receptors (CARs or ‘Frankenstein-cell therapy’) [29,30], their toxicity and complexity of management and manufacturing make this therapy limited and currently only available in selected centers. For this reason, our finding is interesting because patient-derived expanded NK cells armed with an antibody may be a reasonable therapeutic strategy, being less toxic and less expensive than the actual CAR T cells (Figures 5 & 6). NK cell [31] or γδ T cell [28] (‘classical warriors’) plus Trike [32] or antibody-based immunotherapies represent an alterative approach to CAR-T cells therapies (‘Frankenstein cell therapy’) [29,30]. Our findings suggest that different treatment strategies with anti-CD20 monoclonal antibodies alone induce a different behavior in peripheral blood NK cells in humans.

Importantly, one NK cell from a patient may be capable of destroying 25 leukemic cells, even in relapsed patients. Unfortunately, our findings suggest that the human body retains only limited numbers of NK cells, and the capability to refill the blood with new NK cells to attack tumor cells is insufficient to maintain full GA101 activity. We suggest that NK-cell levels may be a predicting factor for the response to obinutuzumab, similar to NK-cell levels in FL [33].

We confirm that during GA101 treatment, the ‘NK reserve’ induces the initial destruction of leukemic cells. We suggest that administration of ‘NK reinforcements’ may be of benefit to patients and should be explored in clinical trials [11,31]. Recently, important and elegant studies about anti-CD20-induced phagocytosis have been published [34,35]. Although other effects of GA101 on Fc-mediated functions elicited by monocytes may be of interest, they may be lower than GA101-induced NK-ADCC [34,35].

### Reflexion about obinutuzumab treatment: the Battle of Thermopylae 

In August of 450 BC, the Persian King Xerxes led a huge army (CLL cells) by land from the Dardanelles, accompanied by a fleet considered to be advancing along the coast. His forces soon reached northern Greece. The alliance of polis, led by Athens and Sparta, tried to stop the advance of the Persians by land, in the narrow pass of Thermopylae, and by sea, in the nearby straits of Artemisio [1].

At the head of the Greek army was the king of Sparta, Leonidas who had about 7000 men (NK and γδ T cells) [36], compared with about 70,000 of the enemy army (CLL cells). The armored infantry of Greece formed a line (NK cells armed with GA101) [22] of few tens of meters of length between a steep hill and the sea. The small battlefield prevented the numerous Persian army (CLL cells) from attacking at once. The Greeks resisted for 2 days with few losses (NK-ADCC induced by first dose of GA101) [7,8], while the Persians suffered many losses (destruction of CLL leukemic burden after first dose of GA101) [22,23]. Xerxes finally discovered a path through which his troops could cross the enemy lines (NK cells-ADCC depletion) [7,8], and on the third day, attacked by that route and swept the flanks of the Greek army (CLL persistence and immune suppression induced in T cells by immunological synapse inhibition or direct destruction) [37–40].

It is said that Leonidas and his troops of about 300 men (NK cells) refused to withdraw because it went against the law and the customs of Sparta, and undertook a last suicide attack that ended their lives (NK cells depletion).

## Conclusion

In summary, despite the low number of patients of this study, our results suggest that the combination of GA101 with NK cells (or NK cells armed *ex vivo*) may help to optimize the GA101-NK ADCC effect and potentially increase the clinical outcome and negative minimal residual disease.

GA101 in monotherapy induces depletion of NK cells in peripheral blood of patients with CLL. NK cells are responsible for most of the tumor burden destruction during initial therapy and their depletion impairs the potential effectiveness of GA101. The combination of GA101 with NK cells may help optimize the potential of this novel anti-CD20 monoclonal antibody. Our observations should be confirmed in large cohorts of patients or clinical trials.

## Future perspective

Although the standard treatment of CLL and FL is currently chemoimmunotherapy, the next years will likely see a shift to chemo-free strategies. New combinations with synergistic mechanisms of action such as monoclonal antibodies plus ‘poisoned apples’ such as BCL-2 inhibitors induce high rates of undetected minimal residual disease, facilitating the work of classical NK and γδ T-cell ‘warriors’ to kill ‘poisoned enemies’ without their survival shields such as BCL-2. Other combinations, such as ibrutinib with their ‘sleeping beauty effect’ [16], may also be effective in combination or in sequential strategies. Although they also induce a lethargic state in the immune system (NK-cell inhibition), monoclonal antibodies such as obinutuzumab may awaken and re-direct the lethargic troops (NK and γδ T cells) to kill sleeping enemies and force them to leave their trenches (lymphocytosis by chemokine-mediated adhesion inhibition). In addition, the ‘sleeping beauty effect’ of B-cell receptor inhibitors is an attractive option when the immune system has been weakened by previous treatments. Last, like the giant creature with super strength, versatility and adaptability created by Victor Frankenstein [30], CAR-T cell therapy has been developed in a similarly expensive and slow creation process but with the power and possibilities to destroy several resistant tumors. However, this powerful strategy may be challenged by a cheap but versatile and faster therapy involving the generation and ex vivo expansion of classical autologous NK or γδ T cell ‘warriors’ re-directed by glycoengineered type II monoclonal antibodies or TriKE against potentially the same tumor antigens of CAR-T cells. In this latest strategy, the monoclonal antibodies or TriKE assumes the function of a general who guides his brave warriors (expansions of LAKs, NK cells or γδ T cells) into battle.

**Summary points**

- Each natural killer (NK) cell of patients with chronic lymphocytic leukemia (CLL) may kill 25 CLL cells.
- Baseline NK-cell count correlates with antileukemic effect.
- One dose of obinutuzumab is able to induce significant reduction of NK cells in peripheral blood.
- In patients with follicular lymphoma, faster recovery of NK cells in peripheral blood was observed during subcutaneous maintenance with rituximab than with intravenous administration after R-CHOP (rituximab–cyclophosphamide–doxorubicin–vincristine–prednisone).
- Patients treated with ibrutinib showed an increment of the NK-cell count after 30 days of treatment in comparison with basal pretreatment levels, but this increment was not statistically significant.
- Therapy with type II anti-CD20 antibodies (obinutuzumab) in patients with CLL simulates a model in which effector cells (NK and γδ T cells) interact directly with target cells (leukemic CD19 B cells) in the blood compartment.
- This model shows that the effector:target ratio is one of the most important factors for maintaining the full effect of immunotherapy and might also be a predictor of response.
